# An Inexpensive, Wearable Patella Reduction Trainer

**DOI:** 10.5811/westjem.2021.12.53140

**Published:** 2022-01-03

**Authors:** Mark Hopkins, Matthew Dalley, Felipe Zinkewich, Ricardo Chujutalli, Deena I. Bengiamin, Timothy P. Young

**Affiliations:** * Loma Linda University Laboratory for Innovations in Medical Education, Loma Linda, California; † Loma Linda University Medical Center, Department of Emergency Medicine, Loma Linda, California

## BACKGROUND

Acute patellar dislocation is a painful condition that can be effectively managed with prompt reduction. Successful reduction requires confidence, which comes with experience. Patellar dislocation is not prevalent enough for every emergency physician to encounter it in a live patient during residency training. Although the reduction maneuver is straightforward, trainees are often initially unsure of hand positioning and attempt to reduce the patella primarily with medial pressure. Simultaneous knee extension is an important component of the reduction, creating patellar and quadriceps tendon laxity and making for a smoother, less painful reduction. Many available videos demonstrate extension poorly and show the difficulty with which the reduction is performed when primarily medial patella pressure is used.^1^,^2^

Simulation is an established modality for teaching procedures. Benefits include learning positioning, approach, and troubleshooting. However, there are currently no commercially produced trainers to teach and learn patellar reduction. Outside of live patient care, we most frequently teach the procedure using the bare, undislocated knee of a volunteer.

## OBJECTIVES

We set out to develop a wearable, low-cost trainer and determine whether the trainer would be preferred to a bare knee as a teaching tool. We undertook this project during a time when COVID-19 limited our ability to gather for learning outside of emergency department (ED) shifts. Consequently, we chose to pilot the trainer on shift and looked to determine the feasibility of this format for future teaching.

## CURRICULAR DESIGN

### The Trainer

We created the trainer from an anatomic knee model (Axis Scientific, Evanston, IL; www.amzn.com/B00KZO8GES). We removed the base and disconnected the patellar tendon (Figure). We used the screw from the base to re-attach the patellar tendon. This allowed the tendon to rotate laterally when the patella dislocates. We attached straps (Magarrow, Guangdong, China; www.amzn.com/B07H19C24Z) to the femur and tibia so that the trainer could be worn on a facilitator’s knee. We needed two additional screws for this, which we had from a previous project. The only tool required was a Phillips head screwdriver. Our total cost for the trainer was $60 and assembly took about 30 minutes.

A reduction with the trainer showing our preferred hand position and technique can be seen here: youtu.be/qi3pHpNjfWc.

### Teaching Session and Data Collection

We aimed to mirror a typical clinical teaching arrangement by pairing a novice with an experienced clinician. Through investigator consensus, we defined an “experienced” clinician as one who had performed three or more live patellar reductions. We defined a “novice” as a clinician who had performed fewer than three live patellar reductions.

We conducted 20 teaching sessions with a single trainer over multiple shifts. One novice and one experienced clinician participated in each session, for a total of 40 participants. All participants provided consent. The experienced clinician used the investigator’s bare knee to teach the novice how to perform a patellar reduction. The novice then performed a patellar reduction on the bare knee. Next, the process was repeated on the opposite knee with the wearable trainer. Last, both the experienced physician and the novice completed a survey. Investigators did not intervene until the session was complete. Sessions lasted 5–10 minutes. Novices did not receive any standardized instruction and experienced physicians were free to teach the procedure as they saw fit.

We developed survey items and responses based on design principles for medical education questionnaires.^3^ We pilot tested the survey among our author group (one medical student, three resident physicians, and two faculty physicians) to improve clarity and functionality but did not collect further survey validity evidence. We sought to compare teaching/learning utility and collect data on the trainer’s realism. These two constructs are commonly evaluated in simulation studies for gathering validity evidence.^4^,^5^ We also planned to measure the trainer’s effect on novice confidence. The survey and study protocol can be viewed here: https://tinyurl.com/6php4a3a.

We compared survey constructs in Stata version 12.1 (StataCorp, LLC, College Station, TX) using a Wilcoxon matched-pairs test. The study was reviewed by our institutional review board, which determined that it did not meet the definition of human subjects research and was exempt from further review.

## IMPACT/EFFECTIVENESS

Survey completion rate was 100%. Experienced physicians rated the trainer higher than the bare knee as a teaching tool, with a median bare knee usefulness of 3/5 (“moderately useful,” interquartile range [IQR] 2.5–4) and a median trainer usefulness of 4/5 (“very useful,” IQR 4–5, *P* = 0.01). Novices rated the trainer higher than the bare knee as a learning tool, with a median bare knee usefulness of 3/5 (“moderately useful,” IQR 2.5–4) and a median trainer usefulness of 4/5 (“very useful,” IQR 4–5, *P* = 0.0004). Experienced physicians rated the feeling of reduction with the trainer as “moderately realistic” (median 3/5, IQR 3–4) and the movements needed to reduce the trainer’s patella as “very realistic” (median 4/5, IQR 3–4). They stated that they would be “very likely” to use the trainer for just-in-time training if it were available during a shift (median 4/5, IQR 4–5).

Novice confidence improved after the session, with a median confidence before the session of 2/5 (slightly confident; IQR 1–3) and a median confidence after the session of 4/5 (very confident; IQR 3.5–5, *P* < 0.0001).

We piloted these sessions on shift and were able to do 20 sessions with 40 participants in about six hours. One strength of an on-shift session is that trainees are already present in the ED. A drawback is that facilitators must dedicate time to organizing the additional sessions. Our routine was to set up the station, announce our presence to faculty, residents, and students on shift and then wait for short breaks in patient care when participation was possible. We recommend scheduling sessions at a time when the ED census is typically lower. We found it helpful to have space available near or in the ED. We have since held similar sessions with other trainers and recommend choosing procedures that can be done quickly. We created subsequent trainers that addressed more complicated joint reductions,^6^ but we found that the simplicity of the patella reduction trainer made for better durability over multiple reductions.

Our study has limitations. Our sample size was relatively small. There is poor agreement regarding how to determine a sample size for studies that evaluate the utility and realism of simulation trainers.^4^,^7^ Our sample size is comparable to that of similar existing studies.^4^ We were able to demonstrate a statistically significant difference in survey constructs with this sample size.

## CONCLUSION

Our patellar dislocation trainer filled an identified, technical skills training need in our program. It was rated as a better teaching/learning tool than a bare knee. We deployed the training on shift, a format that we plan to continue moving forward. The low cost of the trainer makes it a feasible just-in-time teaching tool.^8^ We hope to evaluate its utility in this context in the future.

## Figures and Tables

**Figure:**
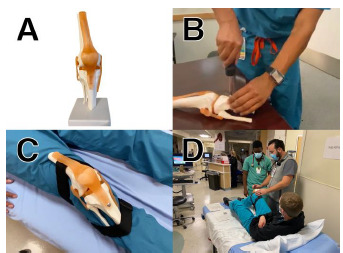
The base of the trainer is an anatomic knee model (A). The “patellar tendon” is removed and reattached with a screw so that the tendon can rotate laterally and allow dislocation (B). Straps are attached to make the trainer wearable (C). We deployed the training in our pediatric emergency department (D).
